# Efficacy and Safety of Albumin-Bound Paclitaxel Compared to Docetaxel as Neoadjuvant Chemotherapy for HER2-Negative Breast Cancer

**DOI:** 10.3389/fonc.2021.760655

**Published:** 2022-01-11

**Authors:** Zhi-Dong Lv, Hong-Ming Song, Zhao-He Niu, Gang Nie, Shuai Zheng, Ying-Ying Xu, Wei Gong, Hai-Bo Wang

**Affiliations:** ^1^ Breast Center, The Affiliated Hospital of Qingdao University, Qingdao, China; ^2^ Department of Breast and Thyroid Surgery, Heze Municipal Hospital, Heze, China; ^3^ Department of Breast Surgery, The First Affiliated Hospital of China Medical University, Shenyang, China; ^4^ Department of Thyroid and Breast Surgery, The Second People’s Hospital of Kunshan, Kunshan, China

**Keywords:** Her-2-negative breast cancer, pathological complete response, neoadjuvant chemotherapy, albumin-bound paclitaxel, docetaxel

## Abstract

**Background:**

Nanoparticle albumin-bound paclitaxel (nab-paclitaxel) as neoadjuvant chemotherapy (NAC) for breast cancer remains controversial. We conducted a retrospective study to compare the efficacy and safety of nab-paclitaxel with those of docetaxel as neoadjuvant regimens for HER2-negative breast cancer.

**Methods:**

In this retrospective analysis, a total of 159 HER2-negative breast cancer patients who had undergone operation after NAC were consecutively analyzed from May 2016 to April 2018. Patients were classified into the nab-paclitaxel group (n = 79, nab-paclitaxel 260 mg/m^2^, epirubicin 75 mg/m^2^, and cyclophosphamide 500 mg/m^2^) and the docetaxel group (n = 80, docetaxel 75 mg/m^2^, epirubicin 75 mg/m^2^, and cyclophosphamide 500 mg/m^2^) according to the drug they received for neoadjuvant treatment. The efficacy and adverse events were evaluated in the two groups.

**Results:**

The pathological complete response (pCR)(ypT0*/*isN0) rate was significantly higher in the nab-paclitaxel group than in the docetaxel group (36.71% vs 20.00%; *P* = 0.031). The multivariate analysis revealed that therapeutic drugs, lymph node status, and tumor subtype were the most significant factor influencing treatment outcome. At a median follow-up of 47 months, disease-free survival (DFS) was not significantly different in those assigned to nab-paclitaxel compared with docetaxel (82.28% vs 76.25%; *P* = 0.331). The incidence of peripheral sensory neuropathy in the nab-paclitaxel group was higher than that in the docetaxel group (60.76% vs 36.25%; *P* = 0.008), while the incidence of arthralgia was observed more frequently in the docetaxel group (57.50% vs 39.97%; *P* = 0.047).

**Conclusions:**

Compared with docetaxel, nab-paclitaxel achieved a higher pCR rate, especially those patients with triple-negative breast cancer or lymph node negative breast cancer. However, there was no significant difference in DFS between the two groups. This study provides a valuable reference for the management of patients with HER2-negative breast cancer.

## Introduction

Neoadjuvant chemotherapy (NAC) has become a treatment option for patients with operable breast cancer (1). NAC performs as a platform to allow time for genetic testing, allow rapid assessment of drug efficacy, and provide important prognostic information (2). After receiving NAC, patients who attained superior pathological complete response (pCR) have been found to be associated with an extremely favorable survival benefit and proposed as a surrogate endpoint for predicting survival outcomes (3).

Taxane-based regimens are widely used in the NAC of human epidermal growth factor receptor 2 (HER2)-negative breast cancer (4). The conventional taxanes include docetaxel and paclitaxel. The NSABP B27 study found that the addition of docetaxel notably increased the pCR rate from 13.7% to 26.1% (5). Docetaxel is extremely hydrophobic and therefore requires a solvent to allow for parenteral administration. Docetaxel is formulated in polysorbate 80 and an ethanol diluent. These solvents are pharmacokinetically active and can cause a number of adverse reactions, such as hypersensitivity reactions and peripheral neuropathy (6). Nanoparticle albumin-bound paclitaxel (nab-paclitaxel) is a unique non-solvent-containing protein formulation. It can obviate the need for prophylactic anti-histamine and steroid treatment because of its much lower risk of hypersensitivity compared with conventional paclitaxel, although it is prone to causing peripheral neuropathy (7). Recently, nab-paclitaxel has been developed and administered to patients with breast cancer (8). Because nab-paclitaxel facilitates the accumulation of a higher paclitaxel dose into cancer cells, it has been expected to exert more feasible effects. Clinical trials of patients with metastatic breast cancer found that nab-paclitaxel achieves longer survival than docetaxel (9). However, evidence is insufficient to judge whether nab-paclitaxel is superior to docetaxel in a neoadjuvant setting. For example, when nab-paclitaxel is administered on days 1, 8, and 15, every 4 weeks, or docetaxel is administered on day 1, every 3 weeks, there is no difference between treatment groups (10). However, another study reported that higher PCR rates were achieved by the nab-paclitaxel group compared with the docetaxel regimen, particularly for the TNBC subpopulation and patients with a high Ki67 level (11). Real-world evidence of nab-paclitaxel as a NAC option for patients with HER2-negative breast cancer is limited (12). The response and adverse event assessments vary in different studies. Thus, to assess the clinical utility of nab-paclitaxel in NAC, we conducted this retrospective study to compare the efficacy and toxicity of nab-paclitaxel-based with those of docetaxel-based regimens used in patients with HER2-negative breast cancer.

## Patients and Methods

### Patients

This is a retrospective study of HER2-negative breast cancer patients who received NAC and underwent surgery at the Affiliated Hospital of Qingdao University. The principle inclusion criterion was (1) age from 18 to 70 years; (2) pathologically confirmed invasive breast cancer; (3) HER2-negative; (4) clinical stage II–III disease; (5) received radical operation for breast cancer; (6) and received taxane–epirubicin–cyclophosphamide (TEC) chemotherapy before surgery. The exclusion criteria for the patients were as follows: (1) received any type of treatment prior to NAC treatment, including chemotherapy, targeted therapy, radiotherapy, or endocrine therapy; (2) with previous or synchronous invasive or *in situ* breast cancer, male breast cancer, bilateral breast cancer, or inflammatory breast cancer; and (3) with acute and chronic inflammatory disease, autoimmune disease, mental disease, severe liver, kidney insufficiency, or serious complications.

**Figure 1:**
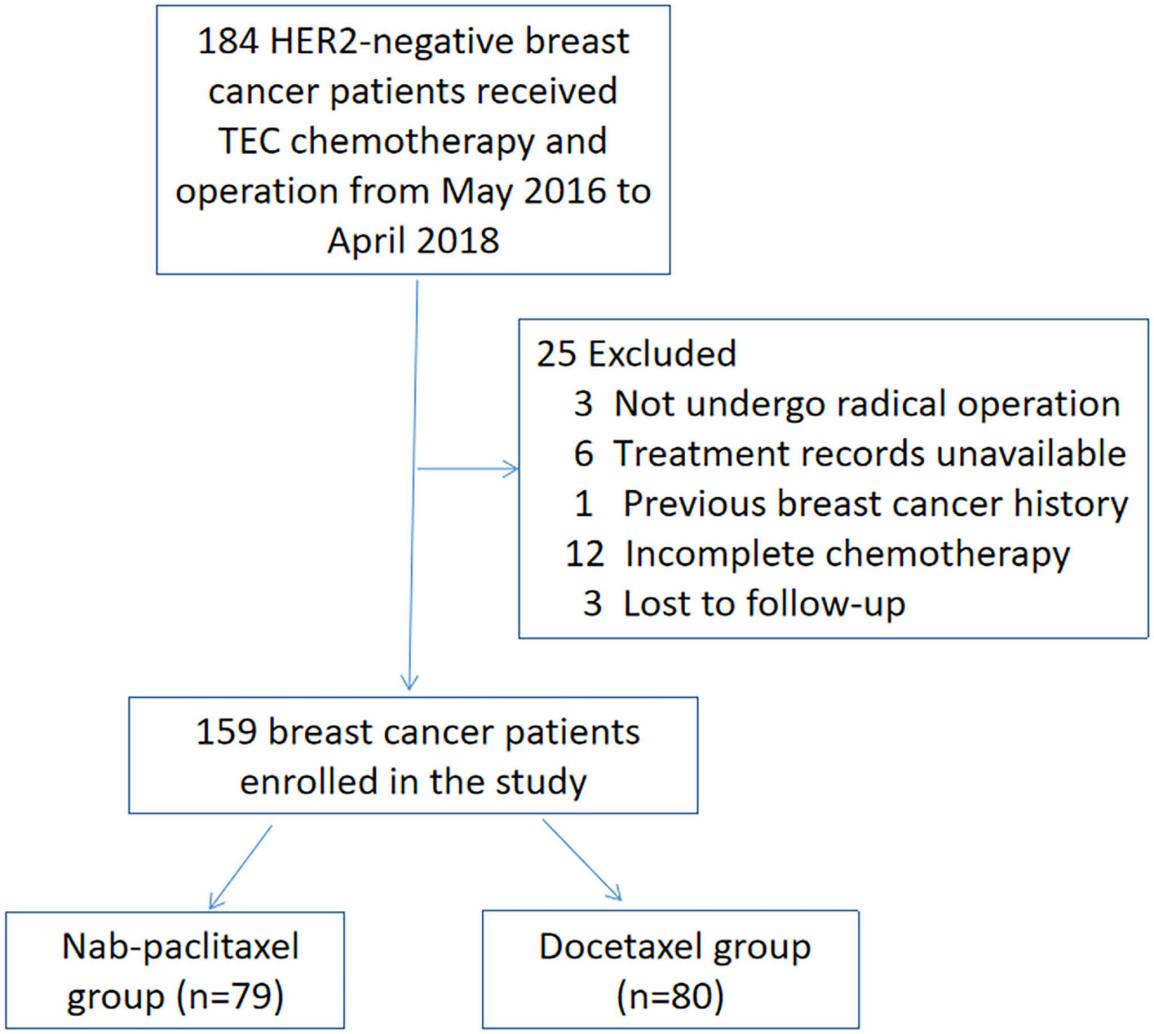
The procedure of screening and grouping patients.

We included all consecutive patients meeting the inclusion/exclusion criteria from May 2016 to April 2018. A total of 184 patients were initially identified. Women who did not undergo radical operation after NAC (n = 3), had treatment records unavailable (n = 6), had previous breast cancer history (n = 1), had incomplete six cycles of chemotherapy (n = 12), or were lost to follow-up (n = 3) were excluded. Finally, 159 breast cancer patients enrolled in the study. The process of screening and grouping is shown in **Figure 1**. Patients were classified into the nab-paclitaxel group (n = 79) and the docetaxel group (n = 80) according to the drug they received for treatment. Patients in the nab-paclitaxel group received six cycles of every 3 weeks (q3w) nab-paclitaxel 260 mg/m^2^, epirubicin 75 mg/m^2^, and cyclophosphamide 500 mg/m^2^. In the docetaxel group, the patients received six cycles of q3w docetaxel 75 mg/m^2^, epirubicin 75 mg/m^2^, and cyclophosphamide 500 mg/m^2^. NAC was delivered according to patients’ specific disease features following the guidelines of the National Comprehensive Cancer Network. The physicians modified the regimen doses and schedules according to the tumor response and side effects. The docetaxel group received intravenous injection of dexamethasone before chemotherapy. A prophylactic injection of granulocyte colony stimulating factor was administered, and all patients underwent surgery 2–4 weeks after NAC.

Pathological diagnosis was obtained *via* core needle biopsy before initiating NAC. Immunohistochemistry (IHC) was used to assess estrogen receptor (ER), progesterone receptor (PR), HER2 status, and Ki-67 level. ER- and PR-positive were defined as ≥1% positively stained tumor cells. HER2 status was evaluated by IHC and fluorescence *in situ* hybridization (FISH). HER2-negative was defined as IHC scoring 1+ or 2+ with FISH non-amplified based on the American Society of Clinical Oncology Guidelines. The cells with Ki-67 were counted and expressed as the percentage of cells with positive nuclear staining among the total tumor cells. The molecular subtypes of breast cancer were classified according to the St. Gallen Consensus. All of the patients were Eastern Cooperative Oncology Group performance status 0–1. This study was approved by the Medical Ethics Committee of the Affiliated Hospital of Qingdao University (QYFY WZLL 26545). Considering the retrospective nature of this work, the requirement for informed consent was waived for individual participants as per the committee standards. To protect the patient’s privacy, we have de-identified all patient details in this paper.

### Response and Toxicity Assessments

Response assessments of NAC by ultrasonography or magnetic resonance imaging were performed within 1 week before NAC and before surgery. The pathology reports were reviewed by two pathologists to determine the pathological response category. The clinical tumor response to NAC was measured using RECIST 1.1. pCR was defined as no pathologic evidence of a residual invasive carcinoma in the breast or axillary lymph nodes (ypT0*/*isN0 status). Residual ductal carcinoma *in situ* was included under pCR. Partial response (PR) was defined as a decline of at least 30% in tumor maximum diameter, and progressive disease (PD) was defined as an increase of at least 20% from the baseline in the sum of all tumor diameter measurements. The disease was categorized as a stable disease (SD) when CR, PR, or PD was not noted. Patients were considered responders if they achieved CR or PR.

The treatment-related adverse events were calculated. Toxicities were graded using the National Cancer Institute Common Toxicity Criteria version 5.0. According to the literature, those effects that were reported to be associated with nab-paclitaxel or docetaxel were examined: leukopenia, thrombocytopenia, peripheral sensory neuropathy, nausea, oral mucositis, cardiotoxicity, rash, and arthralgia. All patients were followed up every 3 months by telephone or outpatient interview for at least 3 years. The disease-free survival (DFS) was calculated as the period from the date of surgery to the first observation of the tumor recurrence (local relapse and/or metastatic recurrence) or the last follow-up. The reporting of this study conforms to STROBE guidelines (13).

### Statistical Analysis

Patient and tumor characteristics and pCR rates were compared between groups by Pearson’s χ^2^ test or Fisher’s exact test. The sample size calculation was performed using the Stata software system (version 14.0, Stata Corp., College Station, TX, USA). Hazard ratios (HRs) and 95% confidence intervals (CIs) were obtained using the stratified Cox proportional hazards model. The Kaplan–Meier method was used to estimate the distributions of survival outcomes. Comparisons in survival rates between the treatment groups were assessed by the logistic regression analysis. The Cox model was used to control intergroup confounding prognostic variables. Statistical Package for Social Sciences (SPSS) (version 19.0, SPSS Inc., Chicago, IL, USA) was used for the statistical analysis, and *P* ≤ 0.05 was considered statistically significant.

## Results

### Clinicopathological Characteristics

From May 2016 to April 2018, 79 patients who underwent nab-paclitaxel-based treatment and the other 80 patients who were administered the docetaxel-based regimens enrolled into the study and were available for analysis. All patients were diagnosed with invasive breast carcinoma using core needle and met the study criteria. The basic clinicopathological characteristics of all the subjects with breast cancer are shown in **Table 1**. The median age was 45 (25–67) years and 47 (27–69) years in the nab-paclitaxel group and docetaxel group, respectively. Baseline characteristics were comparable between the two groups, including age (*P* = 0.940), tumor size (*P* = 0.474), grade (*P* = 1.000), lymph node status (*P* = 0.084), tumor subtype (*P* = 0.822), Ki-67 level (*P* = 0.599), and clinical stage (*P* = 0.580).

**Table 1:** Baseline characteristics of patients.

Subgroup	Nab-paclitaxel (n = 79)	Docetaxel (n = 80)	χ^2^	*P*
Age (years)				
<50	41	43	0.006	0.940
≥50	38	37		
Tumor size			0.512	0.474
T1–2	36	42		
T3–4	43	38		
Grade			0.000	1.000
I–II	39	40		
III	40	40		
Lymph node status			2.976	0.084
Negative	25	37		
Positive	54	43		
Hormone receptor			0.051	0.822
Negative	24	22		
Positive	55	58		
Ki-67			0.276	0.599
≤20%	16	20		
>20%	63	60		
Clinical stage			0.307	0.580
II	38	43		
III	41	37		

### Efficacy

**Figure 2:**
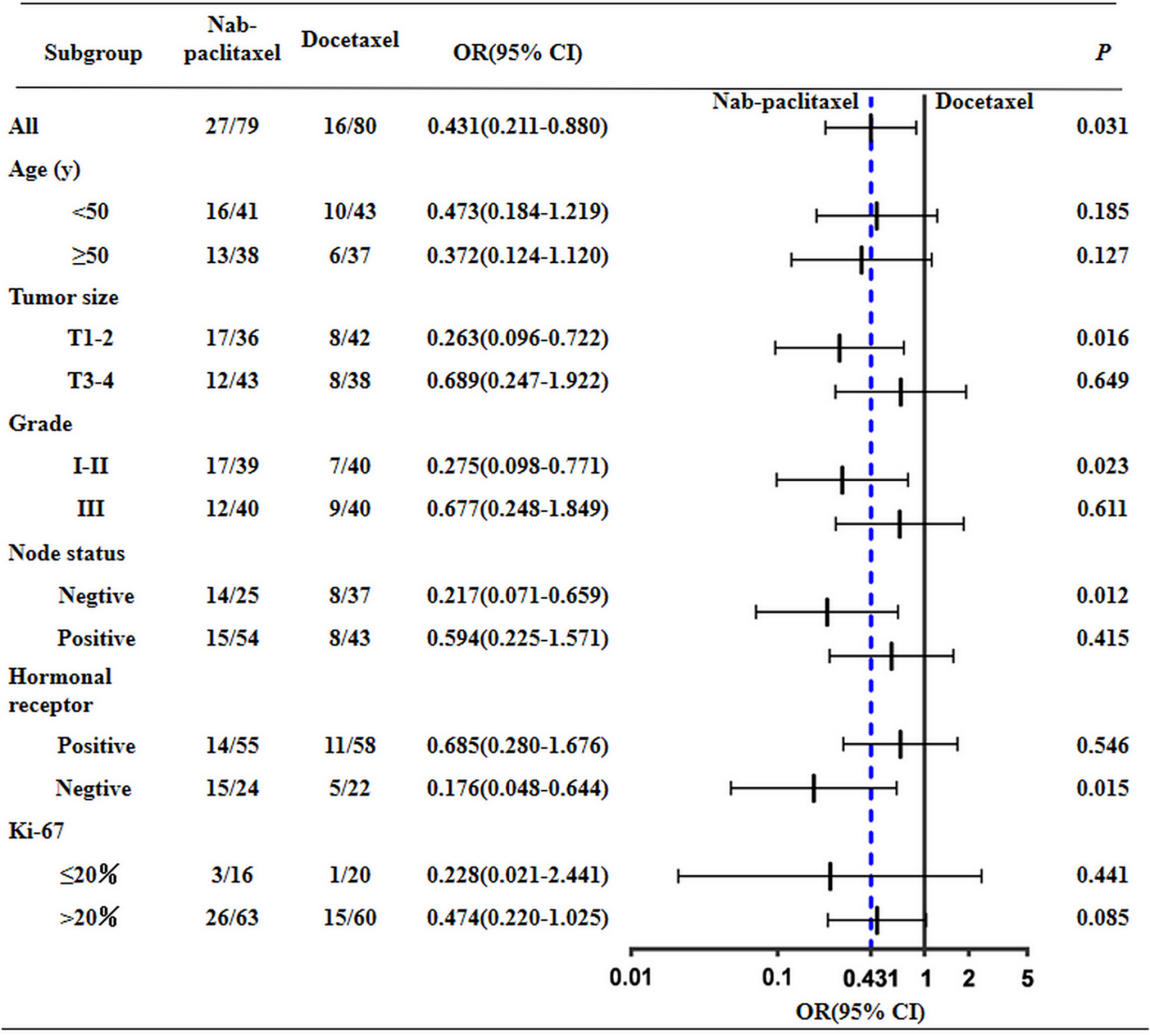
Subgroup analysis of pathological complete response rate.

The overall response rates of the nab-paclitaxel and docetaxel groups were 89.87% (71/79) and 85.00% (68/80); the difference was not statistically significant (*P* = 0.492). After NAC, the pCR rate of the nab-paclitaxel regimens was 36.71% (29/79), which was higher than the rate of 20.00% (16/80) for the docetaxel regimens; the difference was statistically significant (*P* = 0.031) (**Table 2**). After subgroup analysis, patients with triple-negative breast cancer (TNBC) the in nab-paclitaxel group achieved a higher pCR rate than in the docetaxel group (62.50% vs 22.30%; *P* = 0.015). Furthermore, nearly half of the patients with T1–2 in the nab-paclitaxel group achieved pCR, which was significantly greater than in the docetaxel group (43.59% vs 19.05%; *P* = 0.023). The pCR rate of patients with lymph node negative in the nab-paclitaxel group was 56.00%, which was significantly higher than that in the docetaxel group (*P* = 0.012) (**Figure 2**). All of the 159 patients received radical surgery after NAC, 12 patients (15.19%) in the nab-paclitaxel group and 7 patients (8.75%) in the docetaxel group received breast-conserving surgery, and the other patients received mastectomy.

**Table 2:** Pathological response.

Pathological response	Nab-paclitaxel (n = 79)	Docetaxel (n = 80)	χ^2^	*P*
			4.676	0.031
pCR	29	16		
Non-pCR	50	64		
			0.473	0.492
CR+PR	71	68		
SD+PD	8	12		

The results of multivariate logistic regression analysis for the pCR are shown in **Table 3**. In the combination analysis, nab-paclitaxel-based regimens displayed a significantly better pCR compared with the docetaxel-based regimens (OR, 2.777; 95% CI, 1.292–5.969; *P* = 0.009). Among the other four parameters, lymph node status (negative vs positive) and tumor subtype (TNBC vs HR+/HER2-) were the significant factor influencing treatment outcome favoring HER2-negative tumors.

**Table 3:** Multivariate analysis of pCR.

Variable	Effect	OR (95% CI)	*P*
Treatment	Nab-paclitaxel vs docetaxel	2.777 (1.292–5.969)	0.009
Tumor size	T1–2 vs T3–4	1.399 (0.658–2.973)	0.382
Grade	I–II vs III	1.068 (0.506–2.254)	0.864
Lymph node status	Positive vs negative	0.459 (0.215–0.979)	0.044
Tumor subtype	HR+/HER2- vs HR-/HER2-	0.375 (0.174–0.808)	0.012

### Disease-Free Survival

**Figure 3:**
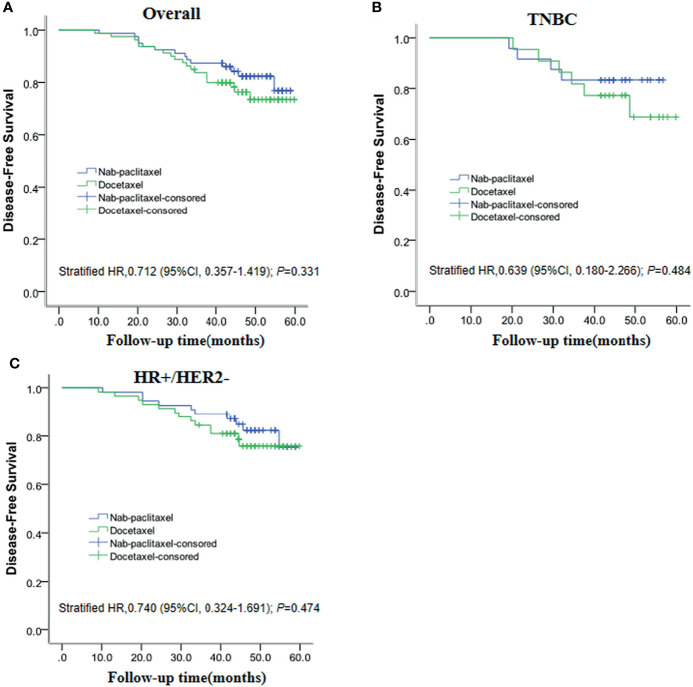
Kaplan–Meier plots show disease-free survival for the entire **(A)**, TNBC **(B)**, and HR+/HER2- **(C)** populations.

The DFS analysis between the nab-paclitaxel group and docetaxel group was examined by using the Kaplan–Meier method with the log-rank test. After a median follow-up of 47 months, 33 of 159 patients (20.75%) experienced DFS events (14 in the nab-paclitaxel group and 19 in the docetaxel group). DFS was not significantly different in those assigned to the nab-paclitaxel group compared with the docetaxel group (82.28% vs 76.25%; *P* = 0.331). At the same time, we found that the DFS of nab-paclitaxel was 83.30% higher than that of 72.70% of docetaxel in TNBC patients, but the difference was not statistically significant (*P* = 0.484). In addition, we found that the DFS of nab-paclitaxel (81.80%) and docetaxel (77.6%) was similar in HR+/HER2- patients (*P* = 0.473). The Kaplan–Meier curves for DFS are depicted in **Figure 3**.

### Safety

Of the 159 enrolled patients in the two groups, all completed six cycles of NAC. During NAC, 94.94% patients in the nab-paclitaxel group had at least one drug-related adverse event compared with 95.00% of those treated with docetaxel. Most of the drug-related adverse events were mild and are listed in **Table 4**. Neutropenia is the most common adverse reaction during chemotherapy; the incidence was 86.08% in the nab-paclitaxel group and 90.00% in the docetaxel group, and there was no statistical significance between the two groups (*P* = 0.420). Peripheral sensory neuropathy (at any grade) occurred more often in patients allocated to the nab-paclitaxel group (60.76% vs 36.25%; *P* = 0.008), whereas the incidence of arthralgia (at any grade) in the nab-paclitaxel group was lower than in the docetaxel group (37.97% vs 57.50%; *P* = 0.047). The other adverse reactions, such as thrombocytopenia, nausea, oral mucositis, cardiotoxicity, and rash, were similar between the two groups, and there was no significant difference (*P* > 0.05).

**Table 4:** Treatment-related adverse events.

Toxicity	Nab-paclitaxel (n = 79)	Docetaxel (n = 80)	χ^2^	*P*
Neutropenia			1.734	0.420
0	11	8		
1–2	42	38		
3–4	26	34		
Thrombocytopenia			2.484	0.289
0	33	43		
1–2	39	30		
3–4	7	7		
Nausea			2.941	0.230
0	15	8		
1–2	37	38		
3–4	27	34		
Oral mucositis			0.802	0.669
0	44	46		
1–2	27	29		
3–4	8	5		
Cardiotoxicity			1.097	0.578
0	62	65		
1–2	16	15		
3–4	1	0		
Peripheral sensory neuropathy			9.700	0.008
0	31	51		
1–2	38	24		
3–4	10	5		
Rash			1.574	0.455
0	65	71		
1–2	12	7		
3–4	2	2		
Arthralgia			6.123	0.047
0	49	34		
1–2	26	39		
3–4	4	7		

## Discussion

Breast cancer has become the first malignant tumor in the world with a high incidence rate (14). NAC, as a platform allowing rapid reduction in tumor size and acquiring of drug sensitivity and prognosis information, has been increasingly employed in breast cancer (15). The gold standard for evaluating the efficacy of NAC is pathological response based on surgical specimens. Patients with pCR to NAC have been found to be associated with an extremely favorable survival benefit and proposed as a surrogate endpoint for predicting survival outcomes (16). Previous studies have revealed an enhanced delivery of nab-paclitaxel to tumors and less toxicity compared with docetaxel (17). As for NAC in early breast cancer, the difference in efficacy between nab-paclitaxel and docetaxel remains controversial. Therefore, we carried out this real-world study to retrospectively evaluate the efficacy and toxicity of the nab-paclitaxel-based and docetaxel-based regimens as NAC for HER2-negative breast cancer.

For nab-paclitaxel, it has been hypothesized that albumin-mediated delivery may result in enhanced transport of nab-paclitaxel to tumors (17) and improved tolerability profile of nab-paclitaxel compared with that of docetaxel at equimolar doses, with shorter infusion schedules and no premedication (18). In a trial of patients with metastatic breast cancer, nab-paclitaxel has been shown to achieve higher response rates and a longer time to progression compared to paclitaxel (9). The safety profiles of nab-paclitaxel were acceptable in most trials (19), but the data of head-to-head comparison between nab-paclitaxel and docetaxel are still lacking. The present study highlights the real-world clinical benefits and adverse event profile of nab-paclitaxel administered as a NAC to patients with HER2-negative breast cancer. In this current study, among the 159 breast cancer patients, the pCR in patients treated with nab-paclitaxel was 36.71%, and pCR in those treated with docetaxel was 20.00%. The GeparSepto trial reported pCR rates in their nab-paclitaxel group of 42.7% for ypT0*/*isN0, which was much higher than our results (20). One possible reason is that our patients had a greater tumor burden, which may have reduced the pCR rates. In the GeparSepto trial, the primary tumor size was about 30 mm and about 45% patients were clinically assessed axillary node stage-positive; in comparison, the tumor size in our study was >40 mm and the proportion of patients categorized as clinically assessed axillary node stage-positive was much higher (68.35%). Furthermore, patients with more aggressive tumors seemed to benefit from nab-paclitaxel (21). Of note, in the GeparSepto trial, the pCR rate almost doubled in the TNBC cohort treated with nab-paclitaxel compared to that for paclitaxel. The present results showed that TNBC patients achieved significantly better pCR rates with nab-paclitaxel than with docetaxel. The pCR rate for patients with TNBC in the nab-paclitaxel group was 62.50%, which is higher than what the ETNA trial reported. In the present study, simultaneous application of taxane–epirubicin–cyclophosphamide may kill tumor cells more quickly and effectively reduce tumor load, while in the ETNA trial, it was the sequential application of those drugs. Another explanation could be that fewer patients enrolled and fewer prognostic events occurred in our analysis, which may affect the results of this study.

pCR is a strong predictor for favorable long-term prognosis in breast cancer. Nevertheless, it remains unclear how large a difference in pCR between nab-paclitaxel and docetaxel can translate into a difference in long-term clinical outcomes. The GeparSepto trial demonstrated that the absolute difference between the two treatment groups needed 20% to result in an improved iDFS (10). After a median follow-up of 47 months, 33 of 159 patients experienced DFS events (14 in the nab-paclitaxel group and 19 in the docetaxel group). Our results also showed that no statistically significant difference was observed for DFS between the nab-paclitaxel group and docetaxel group, though a trend of improved DFS was noted for nab-paclitaxel (82.28% vs 76.25%; *P* = 0.331). TNBC patients achieved a better pCR rate; we further analyzed the prognosis of those patients. Results of TNBC patients in DFS still showed a trend to favor nab-paclitaxel (83.30% vs 72.70%), but no statistical significance was found. This is consistent with the finding from the GeparSepto trial (10). Although in general, individual patients with a pCR also have an improved DFS, on a study level a pCR increase does not always translate into a significantly better long-term outcome. With the development of precision medicine, individual patient data with more molecular information might help dig deeper into the benefit population of nab-paclitaxel in the future.

Previous studies have demonstrated that nab-paclitaxel has almost identical toxicities as conventional taxanes except peripheral sensory neuropathy (22). In contrast to docetaxel, nab-paclitaxel does not utilize non-ionic surfactants to solubilize paclitaxel, which are known to contribute to toxicity and entrap paclitaxel within solvent-based micelles (23). Perhaps because nab-paclitaxel delivery is not complicated by solvents, a higher dose can be administered relative to docetaxel. TEC was a chemotherapy regimen with serious side effects, and leukopenia was the most common adverse reaction. In addition, allergy and vomiting were also common adverse effects. The toxicity profile in the present study was similar to that reported by the GeparSepto (20) and ETNA trials (21). Peripheral sensory neuropathy was more common in the nab-paclitaxel group, while neutropenia was common in the docetaxel group. In the GeparSepto trial, after dose amendment of nab-paclitaxel from 150 to 125 mg/m^2^ continuous weekly for 12 weeks, the frequency of grade 3–4 peripheral sensory neuropathy in the nab-paclitaxel group decreased from 15% to 8% (24). In addition, nausea, arthralgia, and rash were comparable between the two groups, which was consistent with the results of previous findings (12). Long-term follow-up would be necessary to identify symptom relief patterns and their impact on quality of life.

This study had some potential limitations. First, this is a retrospective study without randomization and it was conducted in a single institution. As a result, there may be the potential selective bias and statistical error. Second, it is a small cohort study, which may affect the effectiveness of the results. In the future, a larger sample to verify the results is necessary. Finally, the follow-up of this study is relatively short, so more studies with long-time follow-up are needed to get a more accurate result.

In summary, our real-world study demonstrated that nab-paclitaxel was an effective cytotoxic drug in NAC for HER2-negative breast cancer, especially for patients with TNBC or lymph node negative diseases. However, there was no significant difference in DFS between the two groups. This study provides a valuable reference for the management of patients with HER2-negative breast cancer.

## Data Availability Statement

The original contributions presented in the study are included in the article/supplementary material. Further inquiries can be directed to the corresponding authors.

## Ethics Statement

The studies involving human participants were reviewed and approved by the ethics and research committee of the Affiliated Hospital of Qingdao University. The ethics committee waived the requirement of written informed consent for participation.

## Author Contributions

Z-DL, H-MS, and H-BW conceptualized and designed the study. Z-HN, GN, and SZ collected and analyzed the data and drafted the paper. SZ, Y-YX, and WG carried out the data analysis. All authors contributed to the article and approved the submitted version.

## Funding

The present study was financially supported by Postdoctoral Science Foundation of China (No.2020M672001), Natural Science Foundation of Shandong Province (ZR2020MH274) and Qilu Health Outstanding Young Talents Project of Shandong Province.

## Conflict of Interest

The authors declare that the research was conducted in the absence of any commercial or financial relationships that could be construed as a potential conflict of interest.

## Publisher’s Note

All claims expressed in this article are solely those of the authors and do not necessarily represent those of their affiliated organizations, or those of the publisher, the editors and the reviewers. Any product that may be evaluated in this article, or claim that may be made by its manufacturer, is not guaranteed or endorsed by the publisher.

## Abbreviations

nab-paclitaxel, nanoparticle albumin-bound paclitaxel; pCR, pathological complete response; DFS, disease-free survival; NAC, neoadjuvant chemotherapy; ER, estrogen receptor; PR, progesterone receptor; HER2, human epidermal growth factor receptor 2; PR, partial response; PD, progressive disease; SD, stable disease; TNBC, triple-negative breast cancer; IHC, immunohistochemistry; HR, hazard ratio; TEC, taxane–epirubicin–cyclophosphamide; CIs, confidence intervals.
